# Long-term disability progression of pediatric-onset multiple sclerosis

**DOI:** 10.1212/WNL.0000000000007647

**Published:** 2019-06-11

**Authors:** Kyla A. McKay, Jan Hillert, Ali Manouchehrinia

**Affiliations:** From the Department of Clinical Neuroscience (K.A.M., J.H., A.M.), Karolinska Institutet; and Centre for Molecular Medicine (A.M.), Karolinska Hospital, Stockholm, Sweden.

## Abstract

**Objective:**

To evaluate long-term disability progression in pediatric-onset multiple sclerosis (POMS) and compare to adult-onset multiple sclerosis (AOMS).

**Methods:**

This was a retrospective cohort study using prospectively collected clinical information from the Swedish MS Registry. Clinical features were compared and Kaplan-Meier and Cox proportional hazards regression were used to assess the risk of reaching sustained Expanded Disability Status Scale (EDSS) 3, 4, and 6 in POMS (multiple sclerosis [MS] onset <18 years) and AOMS (MS onset ≥18 years).

**Results:**

A total of 12,482 persons were included; 549 (4.4%) were classified as POMS. The POMS cohort took longer to reach all 3 disability milestones from their MS onset, but did so at a younger age than the AOMS cohort. Primary progressive course (hazard ratio [HR] 4.63; 95% confidence interval [CI] 1.46–14.7), higher relapse rate in the first 5 years of disease (HR 5.35; 95% CI 3.37–8.49), and complete remission from the initial relapse (HR 0.41; 95% CI 0.18–0.94) were associated with an altered risk of progression to EDSS 4 among POMS cases. The same pattern emerged for the risk of reaching EDSS 3 and 6.

**Conclusions:**

Patients with pediatric-onset MS follow a distinctive clinical course, which should be considered in the treatment and management of the disease.

Multiple sclerosis (MS) is a chronic disorder of the CNS that typically presents in early adulthood; however, a minority of patients develop the disease in childhood.^1^ Though pediatric-onset MS (POMS) accounts for only about 2%–10% of all cases, it is particularly distressing to patients and their families.^2^ Persons who develop MS early in life appear to be vulnerable to heightened inflammation and axonal loss.^3^ At the same time, their younger age may provide protection through the brain’s enhanced compensatory abilities.^4,5^

The majority of studies on the clinical course of pediatric MS have been limited by small sample sizes and relatively short follow-up periods.^1^ As such, there remain unanswered questions regarding the long-term prognosis for this subset of patients. We aimed to evaluate the clinical characteristics and neurologic disability progression of a cohort of POMS patients and compare them to cases of adult-onset multiple sclerosis (AOMS) using nationwide information from the Swedish MS Registry.

## Methods

We conducted a retrospective cohort study using prospectively collected clinical information from the Swedish MS Registry (SMSreg). All 64 neurology clinics in Sweden contribute MS-specific clinical information to this resource, which is estimated to capture 80% of all cases of MS in the country.^6^ Registered cases of definite MS with an onset of disease between January 1, 1975, and December 31, 2014, were included, to allow sufficient follow-up time before the study end date of April 15, 2018. Persons with fewer than 2 Expanded Disability Status Scale (EDSS) measurements on record were excluded. Pediatric-onset cases were defined as those whose MS onset occurred before the age of 18 years, in accordance with the definition proposed by the International Pediatric MS Study Group.^7^

Information in the SMSreg includes sex, date of birth, date of MS onset and diagnosis, date and reason for withdrawal from registry (i.e., emigration or death), clinical course (relapsing-onset or primary progressive), and geographical region of residence.^6^ The date of MS onset was defined as the date of first recorded clinical manifestation of MS, and the diagnosis was performed by a neurologist based on the prevailing diagnostic criteria at the time of diagnosis.^8–11^ The SMSreg is a Quality Register that is routinely assessed for accuracy, such that if a person is found to be misdiagnosed, they are removed from the Registry. Detailed information on disease-modifying therapy (DMT) use (product name, start and stop dates), relapses (date of onset and complete remission [complete clinical recovery within 6 months] or incomplete remission), and EDSS scores were prospectively recorded by the treating neurologist. Three distinct endpoints were analyzed: time to EDSS 3 (moderate disability, though fully ambulatory), EDSS 4 (limited walking ability, but able to walk more than 500 m without aid or rest), and EDSS 6 (ability to walk with unilateral support no more than 100 meters without rest). The local Multiple Sclerosis Severity Score (MSSS)^12^ was also calculated to compare disease severity at the first recorded EDSS between groups. DMTs were categorized as first- or second-line based on the prescribing guidelines in Sweden. First-line therapies included interferon-β, glatiramer acetate, teriflunomide, and dimethyl fumarate. Second-line therapies included fingolimod, daclizumab, rituximab, mitoxantrone, and natalizumab. Average annualized relapse rates were calculated for individuals in the first 5 years of disease (from MS onset). CSF results are recorded prospectively in the SMSreg, and were assessed for the cases of pediatric-onset MS who were identified as progressive from onset. Follow-up extended from birth or MS onset until the last available EDSS score prior to emigration, death, or the study end date, whichever came first.

### Statistical analysis

Demographic and clinical characteristics were compared between POMS and AOMS using Pearson χ^2^ for categorical variables and the Wilcoxon rank-sum or Student *t* test for continuous variables. Kaplan-Meier and Cox proportional hazards regression were used to assess the risk of reaching sustained EDSS 3, 4, and 6 (expressed as hazard ratios [HR] with 95% confidence intervals [CI]) in POMS and AOMS. Cox models were adjusted for sex, disease course at onset (relapsing-onset or primary progressive), and time-varying DMT exposure, categorized as none, first-line, or second-line. To address the issue of interval censoring (when an individual reaches the disability milestone in the interval between clinical visits, but the exact date is not known), we ran a complementary analysis using the Weibull model. The Weibull model is equivalent to a Cox model; instead of the baseline hazard being nonparametric, it is assumed to follow a Weibull distribution. By using this methodology, we do not exclude persons who have reached the outcome at their first recorded EDSS, thereby reducing potential bias due to left-censoring.

We used Cox models to explore the influence of clinical and demographic characteristics on time from MS onset to EDSS 3, 4, and 6 exclusively in the POMS cohort. Statistical analyses were performed using R: A Language and Environment for Statistical Computing v.3.4.1 (R Foundation for Statistical Computing, Vienna, Austria; 2017).

### Standard protocol approvals, registrations, and patient consents

The study was approved by the Regional Ethical Review Board of Stockholm, and informed consent was provided from patients for the collection of their clinical information.

### Data availability statement

Data related to the current article are available from Jan Hillert, Karolinska Institutet. To be able to share data from the Swedish MS Registry, a data transfer agreement needs to be completed between Karolinska Institutet and the institution requesting data access. This is in accordance with the data protection legislation in Europe (General Data Protection Regulation [GDPR]). Persons interested in obtaining access to the data should contact Ali Manouchehrinia (ali.manouchehrinia@ki.se).

## Results

Of 14,491 MS cases registered in the Swedish MS Registry with an onset between 1975 and 2014, 12,482 (86.1%) had at least 2 EDSS scores recorded and were included in the analysis. Within this cohort, 549 (4.4%) individuals were classified as pediatric-onset, and 11,933 (95.6%) as adult-onset MS. Age at disease onset of the pediatric cases ranged from 5 to 17 years. Most cases reported an onset in adolescence, but 71 (12.9%) occurred prior to the age of 13, and 15 (2.7%) prior to the age of 10. The female:male ratio for those with an MS onset before age 13 was 1.84, while it was 2.62 for those born between ages 13 and 17, inclusive. This difference was not statistically significant (χ^2^ = 1.75, *p* = 0.19). The majority of cases had a relapsing-onset disease course (98.0%), and most (520; 94.7%) had received a DMT at some point during the follow-up period.

There were no differences in sex ratio between the POMS and AOMS cohorts (table 1). The POMS cohort had a longer diagnostic delay (time between onset and diagnosis of MS) and were more likely to have a relapsing-onset disease course and reside in the North of Sweden relative to the AOMS cases (table 1). The POMS cases had a higher relapse rate in the first 5 years than the AOMS cohort (table 1). Detailed information on the initial event (relapse) was available for a subset of the cohort—168 POMS and 3,462 AOMS. These patients were more likely to be female and reside in South or Central Sweden than persons without records of their initial event (data not shown). Individuals with pediatric-onset MS were more likely to experience complete remission from the initial event than adult-onset patients (*p* < 0.0001, table 1).

**Table 1:**
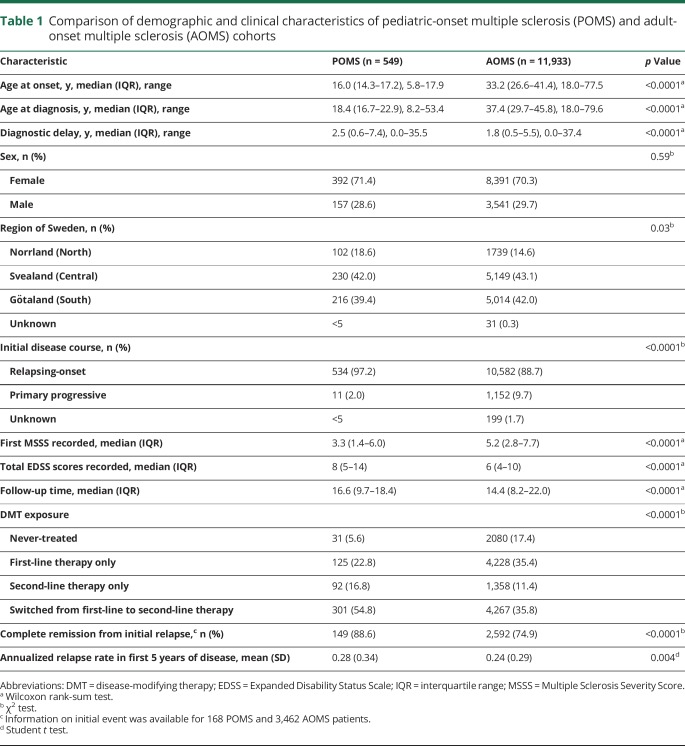
Comparison of demographic and clinical characteristics of pediatric-onset multiple sclerosis (POMS) and adult-onset multiple sclerosis (AOMS) cohorts

Two percent (11/549) of the POMS patients were recorded as having progressive MS at disease onset. Given the rarity of this phenomenon, we explored this group of patients in greater detail. The female:male sex ratio was 0.83, the median onset age was 16.5 (interquartile range [IQR] 14.9–17.1), and the median first recorded EDSS score was 4.0 (IQR 3.0–7.5). Seven of the POMS patients with primary progressive MS (PPMS) had a lumbar puncture performed; all were positive for oligoclonal bands in their CSF. Nine patients showed consistent progression with no evidence of remission (i.e., the EDSS steadily increased over time) for the duration of follow-up (median follow-up 21.8 years from onset [IQR 15.8–23.7]).

**Figure 1:**
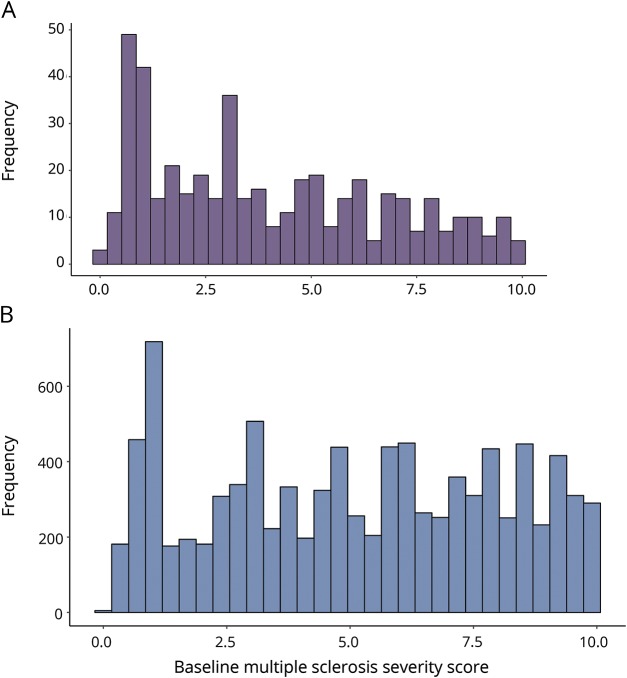
Multiple Sclerosis Severity Scores Histogram of Multiple Sclerosis Severity Scores based on the first recorded Expanded Disability Status Scale from the (A) pediatric-onset and (B) adult-onset multiple sclerosis cohorts.

**Figure 2:**
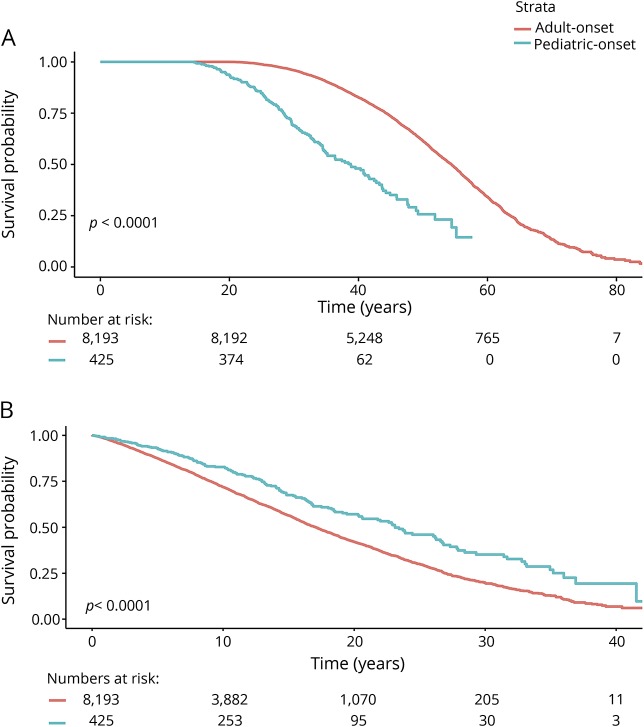
Kaplan-Meier survival curves of time from birth and multiple sclerosis (MS) onset to Expanded Disability Status Scale (EDSS) 3 in pediatric-onset multiple sclerosis (POMS) vs adult-onset multiple sclerosis (AOMS) Time from (A) birth and (B) MS onset to EDSS 3 in POMS vs AOMS.

**Figure 3:**
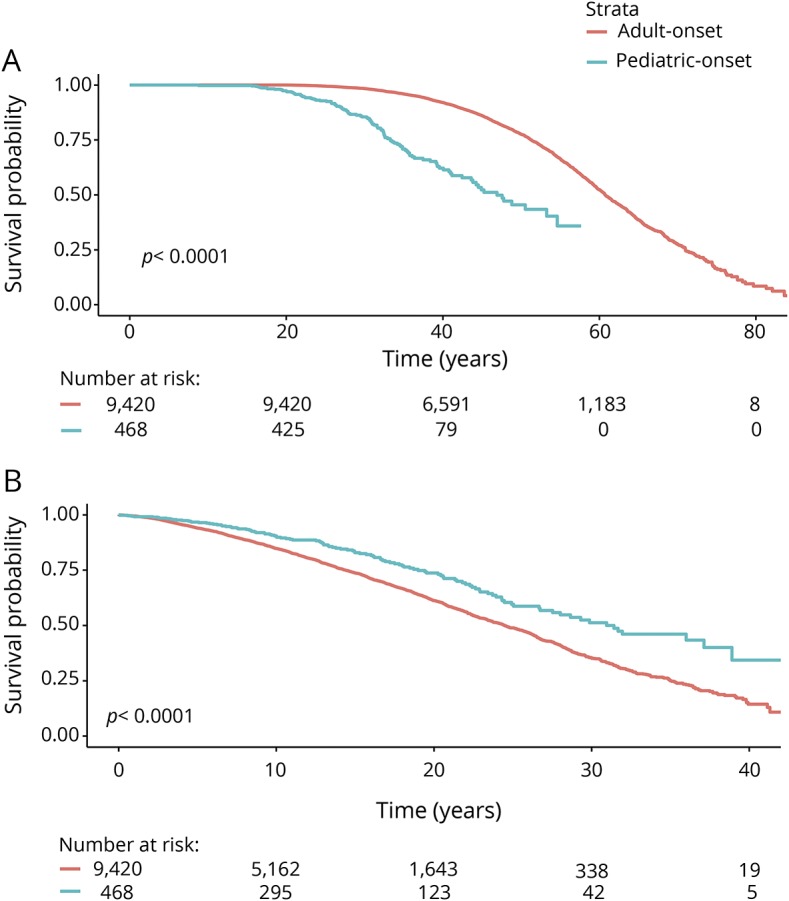
Kaplan-Meier survival curve of time from birth and multiple sclerosis (MS) onset to Expanded Disability Status Scale (EDSS) 4 in pediatric-onset multiple sclerosis (POMS) vs adult-onset multiple sclerosis (AOMS) Time from (A) birth and (B) MS onset to EDSS 4 in POMS vs AOMS.

**Figure 4:**
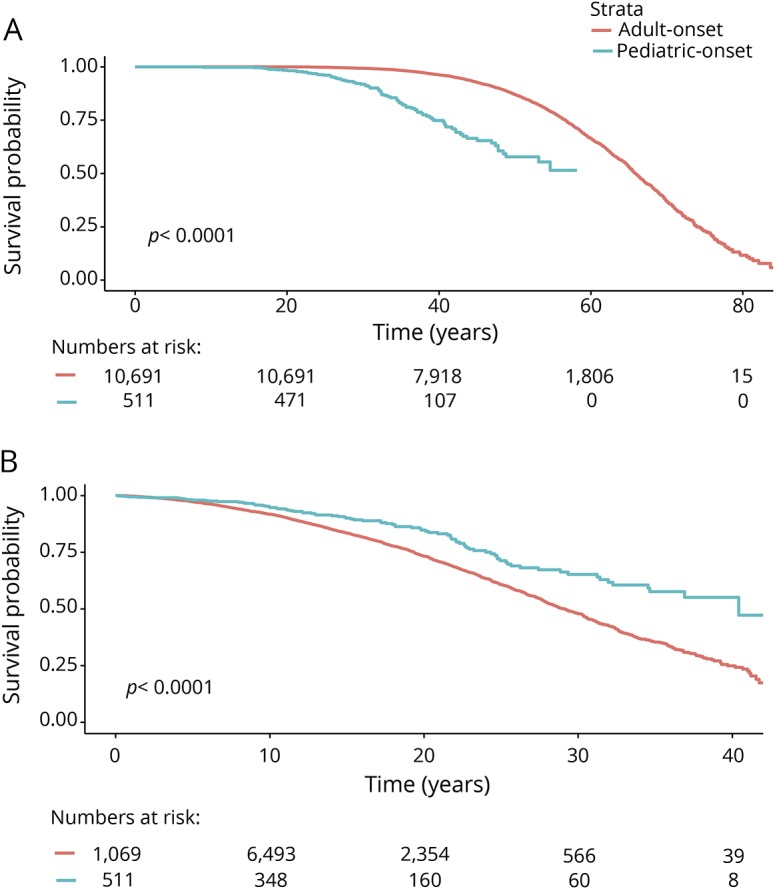
Kaplan-Meier survival curve of time from birth and multiple sclerosis (MS) onset to Expanded Disability Status Scale (EDSS) 6 in pediatric-onset multiple sclerosis (POMS) vs adult-onset multiple sclerosis (AOMS) Time from (A) birth and (B) MS onset to EDSS 6 in POMS vs AOMS.

The median local MSSS based on the first recorded EDSS score was 3.3 (IQR 1.4–6.0) for POMS and 5.2 (IQR 2.8–7.7) for AOMS (*p* < 0.0001, table 1 and figure 1). The median age at which POMS cases reached EDSS 3 was 38.8, compared to 54.4 years for AOMS. The POMS cases took longer to reach EDSS 3 than the AOMS cases from MS onset (23.1 vs 17.1 years, *p* < 0.0001, figure 2). The median age at which POMS patients reached EDSS 4 was 46.9 years, compared to 60.8 years among AOMS (*p* < 0.0001, figure 3). From MS onset, the median time to reach EDSS 4 was longer in the POMS than the AOMS group (31.0 vs 24.5 years, *p* < 0.0001, figure 2). The same pattern emerged for time to EDSS 6; however, less than half of the POMS cohort reached EDSS 6, so an estimate of the median time could not be established (figure 4).

The risk of reaching all 3 disability milestones from birth was higher among POMS compared to AOMS (sustained EDSS 3: HR 2.80; 95% CI 2.33–3.38; sustained EDSS 4: HR: 3.39; 95% CI: 2.74–4.19; sustained EDSS 6: HR 3.67; 95% CI 2.88–4.67). The risk of reaching disability milestones from onset was lower in the POMS cohort relative to the AOMS (sustained EDSS 3: HR 0.77; 95% CI 0.66–0.89; sustained EDSS 4: HR 0.78; 95% CI 0.65–0.95; sustained EDSS 6: HR 0.77; 95% CI 0.62–0.96). All analyses were adjusted for sex, disease course at MS onset, and DMT exposure (table 2). The Weibull models produced the same pattern of results, albeit the risk estimates were less robust (table 3).

**Table 2:**
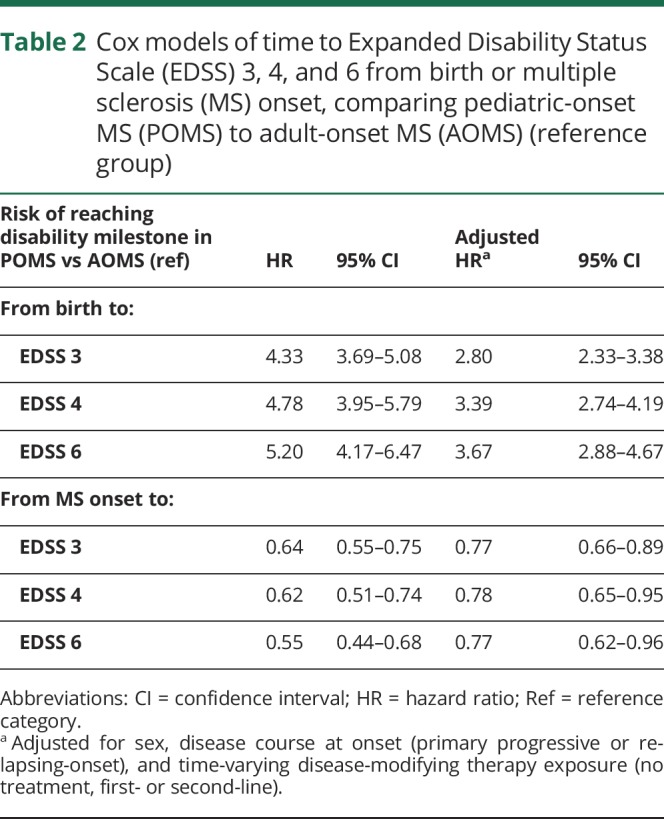
Cox models of time to Expanded Disability Status Scale (EDSS) 3, 4, and 6 from birth or multiple sclerosis (MS) onset, comparing pediatric-onset MS (POMS) to adult-onset MS (AOMS) (reference group)

**Table 3:**
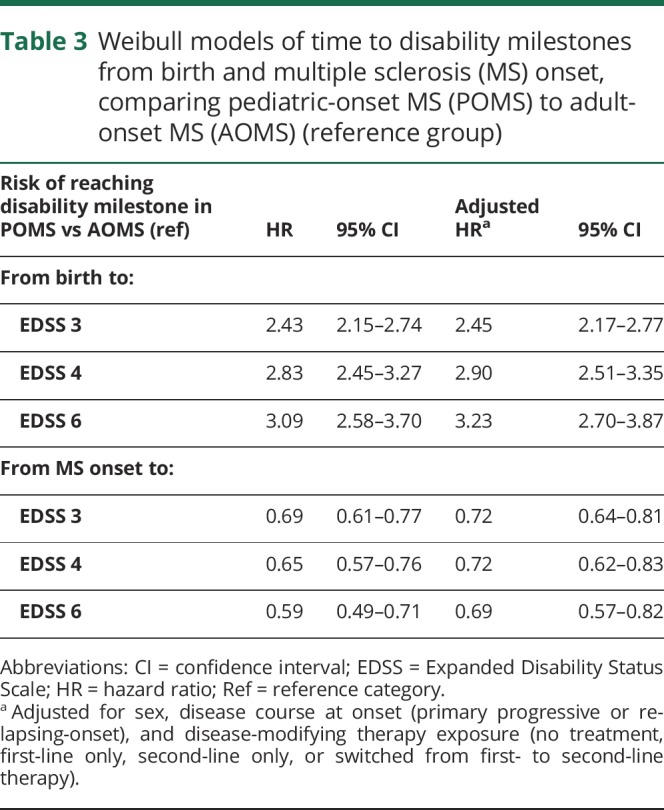
Weibull models of time to disability milestones from birth and multiple sclerosis (MS) onset, comparing pediatric-onset MS (POMS) to adult-onset MS (AOMS) (reference group)

Neither sex nor onset age influenced the risk of reaching any of the 3 disability milestones within the POMS cohort (table 3). POMS with PPMS were at a significantly increased risk of reaching EDSS 4 and 6. Persons with a higher annualized relapse rate (ARR) in the first 5 years of disease were at an increased risk of reaching all 3 disability milestones. Experiencing complete remission from the initial relapse was associated with a reduced risk of reaching EDSS 3, 4, and 6. Second-line DMT exposure did not alter the time to reach disability milestones, but first-line DMT exposure was associated with an increased risk of reaching all 3 (relative to no DMT exposure, table 4). In the multivariate analyses, which included sex, age at onset, disease course, and ARR, exposure to first-line DMTs was found to have no effect on disability, while exposure to second-line DMTs was associated with a reduced risk of reaching EDSS 3 and 4 among POMS (table 5).

**Table 4:**
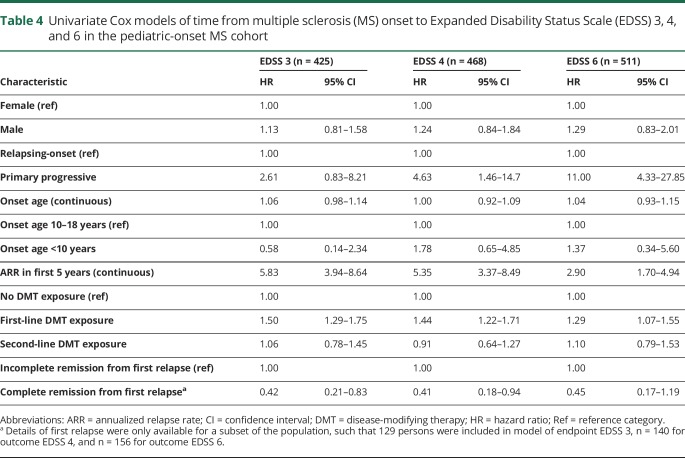
Univariate Cox models of time from multiple sclerosis (MS) onset to Expanded Disability Status Scale (EDSS) 3, 4, and 6 in the pediatric-onset MS cohort

**Table 5:**
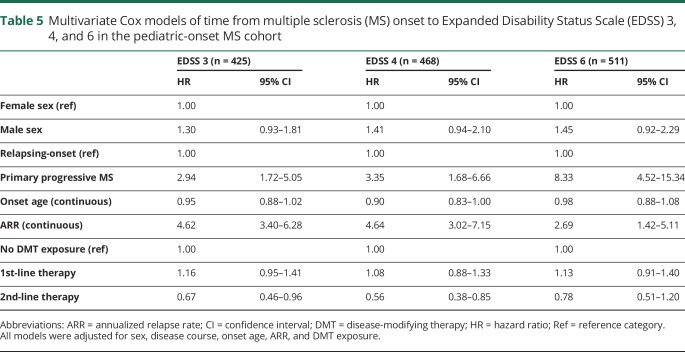
Multivariate Cox models of time from multiple sclerosis (MS) onset to Expanded Disability Status Scale (EDSS) 3, 4, and 6 in the pediatric-onset MS cohort

## Discussion

We identified 549 cases of pediatric-onset MS, representing 4.4% of all registered cases of MS in Sweden. The pediatric-onset cases had a slower disability progression than the adult-onset cases from disease onset, but nonetheless reached disability milestones of sustained EDSS 3, 4, and 6 at a significantly younger age. The POMS cases had an extended diagnostic delay, active early disease, and a high proportion of relapsing-onset disease course relative to AOMS. Among the POMS cases, having a progressive course at onset or a high number of relapses in the first 5 years of disease was associated with an increased risk of disability, while complete remission from the initial relapse was associated with a reduced risk.

Most cases of POMS began in adolescence, but the disease occurred as early as age 5. Having MS from childhood (<10 years) had no measurable effect on the longer-term trajectory of disease, though only 15 persons were in this category, therefore the analysis may have been underpowered to detect a difference. While the vast majority had relapsing-onset MS, 11 cases (2.0%) had a primary progressive disease course. This proportion is in line with findings from other POMS cohorts.^13,14^ Children with PPMS were at a substantially increased risk of MS disability progression; the HR of reaching EDSS 6 was 11.0 relative to relapsing-onset POMS, a much higher risk than that seen in progressive MS in the adult-onset population.^15^ We could not confirm these diagnoses through medical chart review as these individuals have been anonymized, and therefore cannot rule out the possibility that they may have been misdiagnosed. However, all of these patients were followed by their neurologist for an average of 20 years, during which time none were reclassified as non-MS or relapsing-remitting MS/secondary progressive MS. As well, the clinical evidence, including oligoclonal bands in the CSF, high proportion of men, and severe disability, is suggestive of a PPMS course. The median onset age of these patients was 16.5; therefore it is plausible that they represent the tail end of the normal distribution of age at onset for persons with PPMS.

The pattern of disability progression in pediatric-onset relative to adult-onset MS is also concordant with previous findings^13,14,16–18^; however, our estimates of the median time to reach disability milestones were substantially higher. For instance, while earlier estimates of the time to reach EDSS 4 ranged from 10.8 to 23.8 years,^13,14,16–18^ we estimated a median time of 31.0 years from MS onset. This disparity may reflect true differences in survival times between cohorts, or it could be due to methodologic differences between studies. The wider literature of predominantly AOMS has also suggested a slowing of disability progression over time.^19,20^ Prior studies of pediatric MS typically had shorter follow-up times and smaller sample sizes, and employed the outcome of time to first reach a disability milestone,^13,14,16–18^ while the current study employed time to sustained EDSS as the endpoint. In comparison to regional and single clinic-based studies, our data likely capture a wider disability range. Finally, our study took place largely during the treatment era of MS; over 95% of the POMS cases had received a DMT at some point during their disease. It is possible that DMT use has contributed to the extended period of time before reaching disability thresholds. For instance, in the multivariate analysis of POMS cases, we found that second-line DMT exposure was associated with a reduced risk of reaching EDSS 3 and 4. We did not have information on adherence, nor did we adjust for confounding by indication, therefore we are not able to draw conclusions regarding treatment effectiveness within this study.

With the exception of the female:male ratio, the POMS cohort differed from the more common AOMS in every feature studied. By definition, the POMS cases were younger at both MS onset and diagnosis, but they also showed a longer diagnostic delay. This could relate to the infrequency of the disease and the need to explore more extensive differential diagnoses among children.^21^ Regardless of the source of this disparity, it highlights a missed opportunity to commence treatment as early as possible for these patients. POMS patients had higher relapse rates in the first 5 years of disease by 0.04 relapses per year relative to the AOMS. While this was statistically significant, the clinical significance of this difference is marginal. The POMS cases were also more likely to reside in the North of Sweden. There is a marked latitudinal gradient of MS, globally,^22^ and low sunlight exposure (and subsequently lower serum vitamin D) has been posited as one of the potential drivers of this phenomenon.^23^ It is possible that children are exposed to a higher burden of environmental risk, which may contribute to their earlier onset, similar to the higher genetic burden that children with MS bear.^24,25^ An alternative explanation is that this finding relates to differences in access to pediatric care across the country. Specialist pediatric care is more common in the more populous areas in South and Central Sweden. It is possible that patients in the North were more likely to be followed in adult neurology clinics from childhood, and therefore included in the SMSreg during the follow-up period.

This study represents one of the largest population-based studies of POMS to date. Even so, MS progression occurs over decades, resulting in a high rate of right-censoring in the analysis of time to EDSS 6. As with all clinical cohorts, there is a level of imprecision regarding when persons truly met disability endpoints as patients typically come to clinic on an annual basis. We do not expect this to have biased our estimates comparing pediatric-onset to adult-onset MS. It is also possible that very severely progressive patients are not well-represented in the register, which may result in an overestimation of the time to disability. The vast majority of patients in the Swedish MS Registry are of Northern European descent, and therefore these findings are mostly generalizable to this group of patients.

Pediatric-onset MS accounted for nearly 5% of the total incident cases of MS in Sweden between 1975 and 2014. POMS differed from adult-onset cases in several ways, including having an increased diagnostic delay and relapse rate and a higher proportion of cases with relapsing-onset MS. While the POMS cohort took longer to reach disability milestones from their MS onset, they did so at a younger age than the AOMS cohort. This information should be used to inform treatment strategies and health services allocation for this subset of patients. In particular, POMS patients with primary progressive disease, active early disease, or incomplete remission from their first relapse should be monitored closely as they are at a substantially increased risk of disability progression over time.

## Glossary

AOMS: adult-onset multiple sclerosis
ARR: annualized relapse rate
CI: confidence interval
DMT: disease-modifying therapy
EDSS: Expanded Disability Status Scale
HR: hazard ratio
IQR: interquartile range
MS: multiple sclerosis
MSSS: Multiple Sclerosis Severity Score
POMS: pediatric-onset multiple sclerosis
PPMS: primary progressive multiple sclerosis
SMSreg: Swedish MS Registry

## References

[1] Waldman A, Ness J, Pohl D, et al. Pediatric multiple sclerosis: clinical features and outcome. Neurology 2016;87:S74–S81. 27572865 10.1212/WNL.0000000000003028 PMC10688072

[2] Chabas D, Green AJ, Waubant E. Pediatric multiple sclerosis. NeuroRx 2006;3:264–275. 16554264 10.1016/j.nurx.2006.01.011 PMC3593440

[3] Pfeifenbring S, Bunyan RF, Metz I, et al. Extensive acute axonal damage in pediatric multiple sclerosis lesions. Ann Neurol 2015;77:655–667. 25612167 10.1002/ana.24364 PMC4523885

[4] Tortorella P, Rocca MA, Mezzapesa DM, et al. MRI quantification of gray and white matter damage in patients with early-onset multiple sclerosis. J Neurol 2006;253:903–907. 16511645 10.1007/s00415-006-0129-8

[5] Rocca MA, Absinta M, Ghezzi A, Moiola L, Comi G, Filippi M. Is a preserved functional reserve a mechanism limiting clinical impairment in pediatric MS patients? Hum Brain Mapp 2009;30:2844–2851. 19107755 10.1002/hbm.20712 PMC6871244

[6] Hillert J, Stawiarz L. The Swedish MS registry: clinical support tool and scientific resource. Acta Neurol Scand 2015;132:11–19. 26046553 10.1111/ane.12425 PMC4657484

[7] Krupp LB, Banwell B, Tenembaum S; International Pediatric MS Study Group. Consensus definitions proposed for pediatric multiple sclerosis and related disorders. Neurology 2007;68:S7–S12. 17438241 10.1212/01.wnl.0000259422.44235.a8

[8] Poser CM, Paty DW, Scheinberg L, et al. New diagnostic criteria for multiple sclerosis: guidelines for research protocols. Ann Neurol 1983;13:227–231. 6847134 10.1002/ana.410130302

[9] McDonald WI, Compston A, Edan G, et al. Recommended diagnostic criteria for multiple sclerosis: guidelines from the International Panel on the diagnosis of multiple sclerosis. Ann Neurol 2001;50:121–127. 11456302 10.1002/ana.1032

[10] Polman CH, Reingold SC, Edan G, et al. Diagnostic criteria for multiple sclerosis: 2005 revisions to the “McDonald Criteria.” Ann Neurol 2005;58:840–846. 16283615 10.1002/ana.20703

[11] Polman CH, Reingold SC, Banwell B, et al. Diagnostic criteria for multiple sclerosis : 2010 revisions to the McDonald Criteria. Ann Neurol 2011;69:292–302. 21387374 10.1002/ana.22366 PMC3084507

[12] Roxburgh RHSR, Seaman SR, Masterman T, et al. Multiple sclerosis severity score: using disability and disease duration to rate disease severity. Neurology 2005;64:1144–1151. 15824338 10.1212/01.WNL.0000156155.19270.F8

[13] Boiko A, Vorobeychik G, Paty D, Devonshire V, Sadovnick D. Early onset multiple sclerosis. Neurology 2002;59:1006–1010. 12370453 10.1212/wnl.59.7.1006

[14] Renoux C, Vukusic S, Mikaeloff Y, et al. Natural history of multiple sclerosis with childhood onset. N Engl J Med 2007;356:2603–2613. 17582070 10.1056/NEJMoa067597

[15] Raghavan K, Healy BC, Carruthers RL, Chitnis T. Progression rates and sample size estimates for PPMS based on the CLIMB study population. Mult Scler 2015;21:180–188. 25070676 10.1177/1352458514541976

[16] Simone IL, Carrara D, Tortorella C, et al. Course and prognosis in early-onset MS: comparison with adult-onset forms. Neurology 2002;59:1922–1928. 12499484 10.1212/01.wnl.0000036907.37650.8e

[17] Derle E, Kurne AT, Konuşkan B, Karabudak R, Banu A. Unfavorable outcome of pediatric onset multiple sclerosis: follow-up in the pediatric and adult neurology departments of one referral center, in Turkey. Mult Scler Relat Disord 2016;9:1–4. 27645334 10.1016/j.msard.2016.06.002

[18] Harding KE, Liang K, Cossburn MD, et al. Long-term outcome of paediatric-onset multiple sclerosis: a population-based study. J Neurol Neurosurg Psychiatry 2013;84:141–147. 23154123 10.1136/jnnp-2012-303996

[19] Tremlett H, Paty D, Devonshire V. Disability progression in multiple sclerosis is slower than previously reported. Neurology 2006;66:172–177. 16434648 10.1212/01.wnl.0000194259.90286.fe

[20] Manouchehrinia A, Beiki O, Hillert J. Clinical course of multiple sclerosis: a nationwide cohort study. Mult Scler 2017;23:1488–1495. 27956559 10.1177/1352458516681197

[21] Hahn JS, Pohl D, Rensel M, Rao S; International Pediatric MS Study Group. Differential diagnosis and evaluation in pediatric multiple sclerosis. Neurology 2007;68:S13–S22. 10.1212/01.wnl.0000259403.31527.ef 17438234

[22] Simpson S, Blizzard L, Otahal P, Van der Mei I, Taylor B. Latitude is significantly associated with the prevalence of multiple sclerosis: a meta-analysis. J Neurol Neurosurg Psychiatry 2011;82:1132–1141. 21478203 10.1136/jnnp.2011.240432

[23] Handel AE, Giovannoni G, Ebers GC, Ramagopalan SV. Environmental factors and their timing in adult-onset multiple sclerosis. Nat Rev Neurol 2010;6:156–166. 20157307 10.1038/nrneurol.2010.1

[24] Masterman T, Ligers A, Olsson T, Andersson M, Olerup O, Hillert J. HLA-DR15 is associated with lower age at onset in multiple sclerosis. Ann Neurol 2000;48:211–219. 10939572

[25] Isobe N, Keshavan A, Gourraud PA, et al. Association of HLA genetic risk burden with disease phenotypes in multiple sclerosis. JAMA Neurol 2016;73:795–802. 27244296 10.1001/jamaneurol.2016.0980 PMC5081075

